# Clinical Performance of Endocrowns in Molars: A Scoping Review

**DOI:** 10.3390/medicina61091562

**Published:** 2025-08-30

**Authors:** Anna Kontakou Zoniou, Konstantinos Tzimas, Eftychia Pappa, Christos Rahiotis

**Affiliations:** Department of Operative Dentistry, National and Kapodistrian University of Athens, 11527 Athens, Greece; annakontakouzoniou@gmail.com (A.K.Z.); kwstastzimas@dent.uoa.gr (K.T.); effiepappa@dent.uoa.gr (E.P.)

**Keywords:** endocrowns, endodontically-treated teeth, biomaterials, operative dentistry

## Abstract

*Background and Objectives*: Endocrowns have emerged as a minimally invasive restorative option in dentistry, aiming to preserve as much of the original tooth structure as possible. This scoping review investigates the success rates, biomechanical performance, and material efficacy of endocrowns for restoring molars, in comparison to conventional post-and-core crowns. *Materials and Methods*: A comprehensive literature review was conducted to identify relevant studies using PubMed and Scopus databases. The search covered publications up to March 2025. All study types focusing on molar restorations were included, except for case reports. Data extraction and screening were performed independently by two reviewers. *Results*: A total of 37 studies fulfilled the eligibility criteria. Eleven systematic reviews examined comparisons between endocrowns and post-and-core crowns, as well as differences in material selection, survival and success rates, and outcomes between molars and premolars. The remaining 26 studies addressed the clinical performance and longevity of endocrowns, with an emphasis on preparation design, adhesive protocols, and mechanical behavior related to material selection. *Conclusions*: Endocrown restorations present a conservative and predictable alternative to post-and-core crowns for molars with extensive coronal damage. However, due to variability in reported outcomes, high-quality randomized clinical trials are crucial for confirming their clinical effectiveness. The development of novel, standardized treatment guidelines would provide clinicians with adequate information to effectively restore endodontically treated teeth (ETT).

## 1. Introduction

Endodontically treated teeth (ETT) are scientifically documented to be more susceptible to fracture than vital teeth [1]. This is attributed to their significant loss of tooth structure, resulting from procedures such as caries removal, access cavity preparation, root canal preparation, irrigation, and the use of intracanal medicaments [1,2,3,4]. This process exposes the tooth to irreversible physicochemical and biomechanical changes, including dentin dehydration, collagen disruption, and reduced microhardness, conditions that compromise both structural integrity and proprioception [1,5]. Therefore, maintaining as much healthy tooth structure as possible is crucial for the long-term survival of ETT [3,6,7]. This statement is endorsed by studies demonstrating that ETTs present an 80% higher risk of restoration failure compared to vital teeth [8].

Clinicians experience challenges when restoring ETT due to the increased functional and parafunctional loadings that occur on posterior teeth, which contribute to a higher risk of restoration failure [1,9]. ETT restored by post-and-core crowns exhibit higher rates of root fractures, particularly when no circumferential ferrule is obtained. Furthermore, the absence of a ferrule in conjunction with the extensive removal of dentin in the core of the root canals facilitates the debonding of fiber-reinforced posts. The presence of short roots and limited restorative space is a factor associated with restoration failures [5,10]. The limitations associated with traditional restorative approaches, along with recent advancements in adhesive dentistry and dental biomaterials, have driven the development of evidence-based alternative modalities for the restoration of endodontically treated teeth (ETT) [2,3,5,9,10,11,12,13,14,15,16,17,18,19,20].

Pissis, in 1995, first introduced the monobloc technique, described as a laboratory-fabricated core and crown unit designed as a single component [17]. This concept was further developed by Bindl and Mörmann in 1999, who used the term “endocrown” to describe a mono-block ceramic crown bonded to a devitalized posterior tooth [21]. The survival of these one-piece endodontic crowns is achieved through macro-mechanical retention, which serves as an anchorage for the restorative material, providing stability, and micro-mechanical retention via adhesive cementation, forming a strong bonding interface between the restoration and the tooth surface while minimizing microleakage [7,11,12,18,22]. Endocrowns have gained prominence as a minimally invasive technique compared to conventional post-and-core crowns, particularly for molars. Their anatomical design promotes minimal invasive preparation [2,12,16,23] and confines catastrophic failures commonly associated with posts [2,3,5,6,9,18,24].

Factors affecting the survival and success rates of endocrowns include, among others, the material type used and the preparation design, with emphasis on the finish line, occlusal reduction, and extension into the pulp chamber. The extent to which each factor influences the performance of endocrowns needs further investigation. While systematic reviews have focused on specific aspects such as materials or survival rates [2,7,11,12,16], the available literature on endocrown restorations remains diverse in scope, methodology, and outcomes. Given this heterogeneity and the emerging nature of adhesive and restorative techniques, a scoping review is warranted to provide a broad mapping of current evidence, clarify key concepts, and identify gaps for future research. The objective of this scoping review is to investigate and summarize the most recent data on the application of endocrowns for restoring severely damaged posterior ETT, focusing on comparing the clinical performance of endocrowns to that of post-and-core restorations, identifying the most appropriate materials and preparation techniques, and highlighting areas where additional research and standardization are needed.

## 2. Materials and Methods

**Figure 1:**
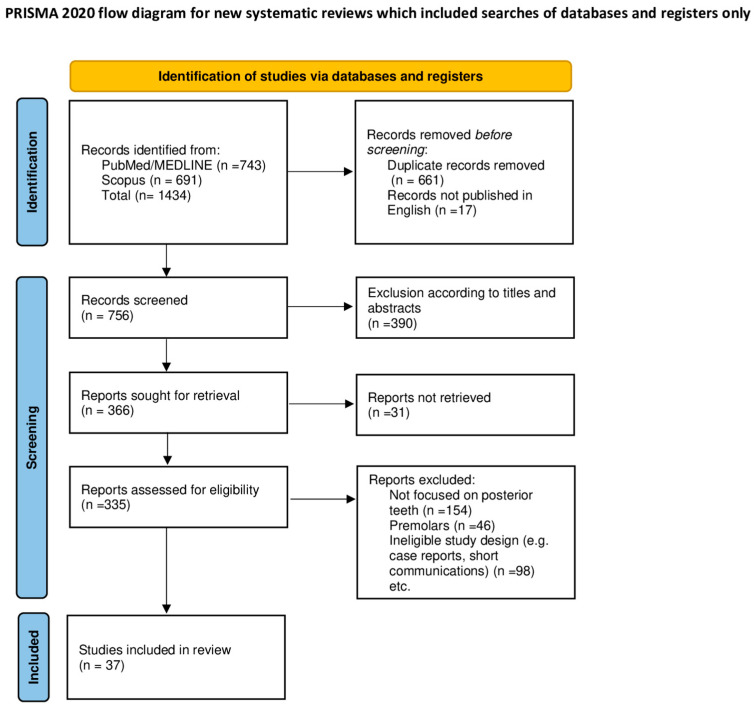
PRISMA-ScR 2020 flow diagram.

**Figure 2:**
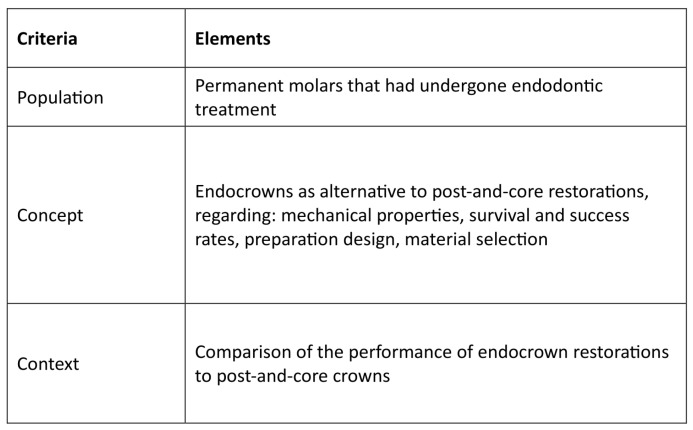
PCC framework.

The reporting of this review adhered to the PRISMA Extension for Scoping Reviews (PRISMA-ScR) checklist [25], thereby enhancing methodological rigor and ensuring systematic reporting. The selection process was documented in a PRISMA 2020 flow diagram (Figure 1) [26], licensed under CC BY 4.0. (https://creativecommons.org/licenses/by/4.0/, accessed on 15 July 2025). The checklist is included in the Supplementary Materials. Although this review protocol was not registered, the PCC (Population, Concept, Context) framework was systematically applied to structure the research question and ensure a transparent selection process (Figure 2).

### 2.1. Sources of Information and Search Strategy

A comprehensive literature search was conducted using the following electronic databases: MEDLINE through PubMed and Scopus, covering publications from their inception to March 2025. While MEDLINE and Scopus were chosen for their broad coverage of dental and medical literature, we acknowledge that additional databases could have provided further studies. Searches in Embase, Web of Science, and Cochrane CENTRAL were considered but not conducted due to resource constraints; however, reference lists of included studies were screened to identify additional relevant publications, thereby reducing the risk of missing important studies.

The search strategy for all databases was conducted through separate queries for each relevant keyword and their Boolean combinations. Queries included “endocrowns”, “endocrowns AND materials”, “endocrowns AND ferrule”, “endocrowns AND survival rate”, and “endocrowns AND post-and-core restorations”. Alternative keywords, including “endodontic crown”, “one-piece crown”, and “monobloc/monoblock” were tested; however, these terms retrieved a plethora of irrelevant publications (e.g., studies on endodontic treatment, one-piece implants, or surgical procedures). Only a restricted number of relevant articles were identified, and these were also captured through other search terms. Additional relevant studies were identified by manually screening the reference lists of all included articles and related reviews. Detailed search strategies for each database, including exact search patterns, applied filters, and date stamps, are provided in Appendix A to ensure transparency and reproducibility.

### 2.2. Eligibility Criteria

The search results were filtered by language, and articles published in English were eligible. The scoping review included systematic reviews, meta-analyses, umbrella reviews, literature reviews, in vitro studies, randomized controlled clinical trials, and retrospective clinical studies. Studies on anterior teeth or premolars were excluded due to differences in their anatomy, occlusal load distribution, and restorative requirements compared with molars, thereby reducing the clinical relevance of comparisons within the context of this review. Case reports, letters to the editor, patents, short communications, and conference abstracts were excluded to maintain clinical relevance, focusing on studies that provide sufficient methodological detail, robust data, and high-quality evidence within the defined scope.

### 2.3. Data Extraction, Screening, and Charting

The study selection process was conducted in two stages. First, titles and abstracts were screened by two independent reviewers (A.K.Z. and K.T.) to identify potentially eligible studies. In the second stage, full-text articles were retrieved and assessed by the pre-established inclusion and exclusion criteria. Disagreements between the two independent reviewers were resolved through discussion. When consensus could not be reached, a third reviewer (E.P.) served as an arbitrator, making the final decision. This procedure ensured transparency and minimized the risk of bias in the selection process. Data charting was performed using a standardized extraction form created and implemented by the reviewers. To ensure consistency, all reviewers pre-tested the form on a sample of studies and refined it through discussion before proceeding with the complete data extraction. Each reviewer independently extracted data, and all charted items were cross-verified to ensure accuracy. The information collected was organized into two Tables. The first table summarizes the results of systematic reviews and meta-analyses, synthesized descriptively. It categorizes them thematically based on comparisons between endocrowns and post-and-core crowns, material selection, and survival and success rates. The second table reported the results of eligible literature reviews, umbrella reviews, in vitro studies, retrospective studies, and RCTs, giving an insight into clinical performance and long-term outcomes of endocrowns, preparation design, cementation processes, mechanical properties, and material selection. The table of systematic reviews and meta-analyses included the author and year of publication, main objectives, search strategy, data screening information, and key findings. The second Table included the author and year of publication, study type, main objective, study design, and key findings. No assumptions or simplifications were made during this process.

Given the nature and purpose of scoping reviews, no formal critical appraisal of the included studies was performed. However, study type, study design, and methodological rigor of each study were considered during data synthesis and interpretation.

## 3. Results

A total of 1434 articles were identified through comprehensive searches conducted in the MEDLINE database via PubMed and Scopus. After record removal due to duplicates, language restrictions, lack of relevance (e.g., anterior teeth), ineligible study types (e.g., case reports, letters, short communications, conference abstracts), insufficient focus on posterior ETT, lack of direct evaluation of endocrowns to post-and-core restorations, or failure to meet design criteria, a total of 37 articles were included in this scoping review. The selection process is outlined in Figure 2 [26].

The included studies were categorized into two primary groups based on study type and are reported in Table 1 and Table 2. All systematic reviews and meta-analyses (11 articles) [2,6,7,11,12,16,23,24,27,28,29] are included in Table 1, which were thematically subcategorized as described in the “Methods and Materials” section.

Table 2 reports twenty original research studies, including fifteen in vitro studies, three RCTs, two retrospective clinical studies, and six reviews (umbrella narrative and literature reviews). These studies were categorized according to their main subject of interest: Ten studies evaluated the clinical performance and long-term outcomes of endocrowns [1,8,10,14,30,31,32,33,34,35], six studies focused on the preparation design and cementation procedure applied [15,18,36,37,38,39], four studies examined the mechanical strength and load resistance influenced by material and preparation design [4,13,40,41], and six studies compared the different materials that are available for endocrown restorations [19,20,42,43,44,45].

**Table 2:** Endocrowns: survival and success rates, indications, preparation design, fracture resistance, and material type.

Author(s)/Publication Year	Study Type	Objective	Study Design	Main Findings/Conclusion
**Clinical Performance and Long-Term Outcomes**				
[35] Otto et al., 2015	Clinical Trial	Evaluation of long-term outcomes of chairside CAD/CAM feldspathic ceramic posterior shoulder crowns and endocrowns	55 patients, with test group: 25 endocrowns (20 molars, five premolars) and control group: 40 shoulder crowns (8 conventional crowns and 32 “reduced prep” crowns) produced using a CAD/CAM system with feldspathic ceramic and examined at baseline and up to 12 years (mean 10 years, 8 months).	Shoulder crowns showed 12-year survival of 95% on molars and 94.7% on premolars. Endocrowns had lower survival rates: 90.5% on molars and 75% on premolars.
[30] Belleflamme et al., 2017	Retrospective Study	Evaluation of ceramic and composite endocrowns with IDS, analyzing failures about tooth preparation and occlusal parameters.	Evaluation of 99 cases with a mean observation period of 44.7 ± 34.6 months using FDI criteria, based on residual tooth tissue and preparation characteristics	Endocrowns are reliable, with a 10-year survival rate of 98.8% and a success rate of 54.9%. They preserve tissue and reduce failures versus post-and-core crowns. IDS increases bonding. LDS ceramics excel, while PICNs require further research.
[31] Fages et al., 2017	Clinical Trial	Determination of the survival rates of chairside CAD/CAM-fabricated all-ceramic crowns and endocrowns for molars in clinical practice.	Between 2003 and 2008, 323 patients received 447 chairside CAD/CAM feldspathic ceramic restorations (212 crowns and 235 endocrowns) on molars by the same dentist, who then followed them up for 7 years.	Endocrowns showed a survival rate of 98.7% and a success rate near 100%. All failures were attributed to partial ceramic fractures within the first two Chairside CAD/CAM all-ceramic crowns and endocrowns provide effective, long-term restorative outcomes.
[1] Alhamdan et al., 2024	Narrative Review	Assessment and comparison of treatment options to provide clinical recommendations for restoring posterior ETT.	PubMed and Google Scholar published between 1977 and 2024.	Direct and indirect restorations have similar survival rates. Decisions depend on the dentist’s experience, tooth structure, ferrule, and restoration properties.
[14] Ciobanu et al., 2023	Literature Review	A comprehensive overview of endocrowns and evaluation of the impact of various materials and preparation designs on their mechanical properties, survival, success rate, and esthetics.	PubMed, Scopus, Web of Science, and Scielo Identified records: 163 Included records: 37	Endocrowns perform similarly or better than other restorations for extensively damaged ETT. LDS and RNC are the most successful materials.
[8] Morimoto et al., 2024	Umbrella Review	Synthesis of evidence from systematic reviews on 1-piece endodontic crowns in posterior teeth, assessing clinical outcomes, survival, success rates, and PROMs, with a null hypothesis of no significant difference from complete crowns.	MEDLINE/PubMed, WOS, Cochrane, OpenGrey, and manually (up to June 2024) Identified records: 468 Included records: 9	Indirect resin and ceramic endocrowns perform similarly to complete crowns with posts. Limited data exist on zirconia and metal crowns, and PROMs remain unaddressed. Low-quality studies, heterogeneity, and overlapping data limit conclusions.
[10] Papalexopoulos et al., 2021	Literature Review	Evaluation of endocrowns as a reliable alternative for extensively damaged ETT, focusing on their indications, contraindications, preparation, and materials.	Review of the literature with keywords “Endocrowns”, “Endodontically treated teeth’, “Literature review”, “Restorative dentistry”	Success rates comparable to conventional crowns (mainly molars). Limited data for premolars/anterior teeth. Retention depends on adhesive cementation. LDS and composite resins show strong bonding.
[32] Fathi et al., 2022	Umbrella Review	Evaluation and comparison of the success rates of various prosthetic restorations on ETT.	MEDLINE/PubMed, Cochrane, and Google Scholar (up to November 2020) Identified records: 43 Included records: 14	Endocrowns and single crowns show similar effectiveness for restoring ETT. No statistically significant differences reported.
[33] Keskin et al., 2024	Clinical Trial	Comparison of the clinical efficacy of RNC and ZLS ceramic endocrowns in treating ETT using a chairside CAD/CAM system.	Ninety endocrown restorations were fabricated in posterior teeth (52 RNC, 38 ZLS) using a CAD/CAM system with a three-year follow-up period.	Survival rates were for RNC 82.7% and for ZLS 86.8%. Both materials are suitable for chairside endocrowns restorations for ETT. No statistically significant differences were found in debonding, ceramic fractures, tooth fractures, and secondary caries.
[34] Kuang et al., 2022	Retrospective Study	Evaluation of the survival rate and clinical performance of CAD/CAM ceramic endocrowns in posterior ETT.	A total of 101 CAD/CAM ceramic endocrowns on posterior teeth were performed on 74 patients from January 2016 to June 2017 and evaluated for their survival rate after 5 years.	5-year survival rate: 93.0% for CAD/CAM ceramic endocrowns. Clinical outcomes:93% showed good anatomic form, and 95% had proper marginal adaptation. Limitations: only 38% showed a good color match with adjacent teeth. No significant differences in survival rates based on sex, tooth position (premolars vs. molars), or materials used.
**Design and Adhesion Considerations**				
[38] Magne et al., 2014	In Vitro Study	Evaluation of the influence of different adhesive core buildup designs on the fatigue resistance and failure mode of endodontically treated molars restored with RNC CAD/CAM crowns using self-adhesive resin cement.	Forty-five human molars were divided into three groups (*n* = 15) based on the restorative technique: Group I: 4 mm adhesive core buildup with complete crown restorations. Group II: 2 mm adhesive core buildup with complete crown restorations. Group III: No adhesive buildup (endocrown restoration). All groups were subjected to a failure test.	Buildup design did not significantly influence the fatigue resistance of RNC CAD/CAM crowns. All designs, including endocrown, survived regular masticatory forces. Failure modes: more favorable in the 2 mm buildup and endocrown groups than in the 4 mm buildup group.
[36] Dartora et al., 2018	In Vitro Study	Comparison of the biomechanical behavior of ETT restored with different endocrown extensions into the pulp chamber.	30 human molars were divided into 3 groups (*n* = 10) based on intracoronal extension depth (5 mm, 3 mm, 1 mm) and loaded to fracture.	Deeper pulp chamber extension: enhances mechanical performance, improving resistance, favoring favorable fracture modes, and optimizing stress distribution.
[15] Einhorn et al., 2019	In Vitro Study	Evaluation of the impact of ferrule inclusion on the fracture resistance of endocrowns specifically for mandibular molars.	Mandibular third molars (*n* = 12/group) were prepared by removing coronal tooth structure and restoring the chamber with resin core material. Ferrule heights were 1 mm, 2 mm, or none for each group. CAD/CAM LDS restorations were placed and subjected to failure testing.	A 1 mm ferrule effect reduced catastrophic failures, but all endocrowns failed at loads exceeding normal masticatory function. Further studies on adaptation and fatigue are needed.
[18] Ribeiro et al., 2023	In Vitro Study	Exploration and characterization of the influence of the height discrepancy between the pulp chamber floor and the crestal bone on the mechanical fatigue performance of ETT restored with resin composite endocrowns.	75 human molars were divided into 5 groups (*n* = 15) based on the pulp chamber floor position relative to crestal bone height (2 mm above, 1 mm above, leveled, 1 mm below, and 2 mm below). All were restored with 1.5 mm-thick composite resin endocrowns and subjected to fatigue failure testing.	The insertion level of the dental element being rehabilitated with an endocrown significantly affects its mechanical fatigue performance. A higher pulp chamber floor relative to the crestal bone increases the risk of mechanical failure. A lower pulp chamber floor height increases the risk of irreparable failures.
[37] Huang et al., 2023	In Vitro Study	Analysis of stress distribution in an endodontically treated mandibular molar with various endocrown configurations, particularly focusing on those with significant defects in the mesial wall.	Four distinct finite element models were constructed based on different endocrown configurations for a mandibular molar. Control Model: butt joint preparation with a 2 mm occlusal thickness. Experimental Models: three butt joint designs with varying distances between the bottom of the mesial wall preparation and the cemento-enamel junction set at 2 mm, 1 mm, and 0 mm, respectively. All models are loaded with vertical and oblique forces.	Increasing simulated defects in the mesial wall elevated peak Von Mises stress in the cement layer, with defects up to the cemento-enamel junction level posing the highest failure risk, particularly in cervical dentin.
[39] Zeng et al., 2024	In Vitro Study	Evaluation of the stress distribution in endocrown restorations applied to ETT, focusing on the effects of different margin designs and loading conditions, and determining how these factors influence stress concentrations and the overall mechanical performance of endocrowns.	Three-dimensional finite element models were created to simulate ETT molars restored with endocrowns. Groups: butt-joint (E0), 90° shoulder (E90), and 135° shoulder (E135) with shoulder group dimensions 1.5 mm height and 1 mm width. Static loads totaling 225 N were applied in 9 locations on the occlusal surface under both buccal and lingual loading conditions.	Stress distribution was similar across the three margins. Shoulder-type designs, particularly the 135° shoulder, demonstrated reduced stress concentration compared to the butt-joint design. Stress levels increased under lingual loading conditions, indicating that loading direction significantly influences stress distribution in endocrowns.
**Mechanical Strength and Load Resistance**				
[13] Biacchi et al., 2012	In Vitro Study	Comparison of the fracture strength of endocrowns and glass fiber post-retained conventional crowns, focusing on their mechanical performance under load to ensure the durability and functionality of ETT.	20 human molars divided into two groups: glass fiber post-retained crowns and endocrowns anchored in the pulp chamber, subjected to controlled loading to assess fracture resistance and failure modes.	Endocrowns demonstrated superior fracture strength compared to glass fiber post-retained crowns.
[41] El Ghoul et al., 2019	In Vitro Study	Evaluation of the fracture resistance and failure modes of endocrowns made of three CAD/CAM materials subjected to thermos-mechanical cycling loading	80 human molars were divided into 4 groups (*n* = 20), (LDS crowns, CAD/CAM LDS endocrowns, CAD/CAM ZLS endocrowns, and CAD/CAM RNC endocrowns) with half of them from each group being loaded axially and the other half laterally until fracture.	RNC, LDS, and ZLS endocrowns have greater fracture resistance than conventional ceramic post-and-core crowns, with LDS performing best under both axial and lateral loading. Irreparable fractures were common (30–70%) across all endocrown groups, highlighting the need for further studies.
[40] Anton Y Otero et al., 2021	In Vitro Study	Evaluation of the fatigue resistance of cracked endodontically treated molars restored with CAD/CAM resin composite endocrowns reinforced with different fiber-reinforced composite bases.	50 human molars were simulated with cracks and divided in 5 groups (group 1: cavity floors lined with 0.5 mm of flowable composite, group 2: cavity floors covered with one layer of FRC-net, group 3: cavity floors covered with three layers of FRC-net, group 4: cavity floors covered with 1 mm of flowable FRC-resin, group 5: cavity floors covered with 2 mm of flowable FRC-resin. Groups 1,2,3 use a different flowable resin composite for the preparation of the chamber than groups 4 and 5, with alterations in composition. All groups submitted to loading testing for fatigue resistance.	Fiber reinforcement did not improve the fatigue resistance of cracked ETT molars with endocrowns. Fiber reinforcement improved the chance of repairability.
[4] Kassis et al., 2021	In Vitro Study	Evaluation of the fracture resistance and failure modes of endodontically treated mandibular molars restored with different designs of inlays, onlays, and endocrowns.	180 human third molars were divided into 6 groups (*n* = 30): control (no preparation), inlay with EverX Posterior, inlay with G-aenial Universal Flo, onlay with EverX Posterior, onlay with G-aenial Universal Flo, and endocrown with an empty pulp chamber and subjected to compressive load.	Endocrowns had the highest fracture resistance, similar to onlays and higher than inlays. Endocrowns had a more favorable failure pattern than inlays, indicating that the design of the restoration influences both fracture resistance and failure patterns.
**Material- Based Comparisons**				
[42] Dartora et al., 2019	In Vitro Study	Comparison of the fatigue performance and stress distribution of endodontically treated molars restored with CAD-CAM endocrowns made from lithium disilicate or polymer-infiltrated ceramic, and with direct composite restorations.	48 human molars were divided into three groups (*n* = 16) and restored with: LD endocrowns, PICN endocrowns, and direct composite restorations. Specimens underwent step-stress fatigue testing, fractographic analysis, and finite element analysis.	LD and PICN endocrowns and direct composite restorations had similar fatigue loads, cycles to failure, and survival probabilities. Indirect restorations, particularly LD, provided higher structural reliability. Stress in tooth tissues was highest for resin composite, lower for PICN, and lowest for LD.
[44] Manziuc et al., 2023	Literature Review	A review of the literature about the mechanical and biological properties of ZLS in CAD/CAM systems.	PubMed, Web of Science, Cochrane, and manually. Identified records: 154 Included records: 71.	ZLS restorations show better mechanical properties than feldspathic, LDS, hybrid ceramics, and RNC, but are inferior to translucent or high-translucency zirconia. Marginal adaptation is almost equal to LDS. ZLS CAD/CAM restorations exhibit the least color change, compared to resin-based materials. ZLS exhibits superior mechanical properties compared to LDS
[20] Veselinova et al., 2023	In Vitro Study	Comparison of the mechanical behavior of ETT restored with endocrowns or overlays made from either monolithic LDS or monolithic zirconia.	48 human molars divided into 4 groups (*n* = 12): overlays restored with monolithic LDS, overlays restored with monolithic zirconia, endocrowns restored with monolithic LDS, endocrowns restored with monolithic zirconia) and subjected to a fracture strength test.	LDS endocrowns have higher fracture strength and greater reliability than monolithic zirconia or overlay restorations. Endocrowns had more catastrophic failures compared to overlays.
[19] Vervack et al., 2024	In Vitro Study	Evaluation of the fracture strength and failure modes of LDS and RNC used as restorations (crown, overlay, or endocrown) on endodontically treated molars.	60 molars were restored with two primary materials LDS and Hybrid Composite. Each material was employed in three-dimensional restoration designs: monolithic endocrown, crown with a separate composite core, and overlay without core buildup or pulpal extension, and subjected to fracture strength test. Ten sound served as a control group.	Fracture loads were similar among all restoration types. Restoration type and material used affected failure modes. All restoration types demonstrated fracture strengths comparable to intact teeth. Endocrowns showed slightly lower fracture resistance compared to crowns and overlays, but within clinically acceptable limits. LDS restorations predominantly showed catastrophic fractures, while RNC restorations had more repairable fractures.
[43] Jalalian et al., 2024	In Vitro Study	Comparison of the fracture resistance and marginal adaptation of CAD/CAM LDS and ZLS endocrowns.	24 human molars were divided into 2 groups (*n* = 12) for ZLS and LDS endocrown fabrication using CAD/CAM. Vertical marginal gap was measured at three stages: before cementation, after cementation, and after thermomechanical cycling. Fracture resistance was tested at a 45° angle, and failure mode was assessed.	ZLS endocrowns exhibited superior marginal adaptation, higher fracture resistance, and more irreparable fractures than LDS endocrowns, Both ZLS and LDS showed acceptable vertical marginal adaptation.
[45] Taha et al., 2024	In Vitro Study	Comparison of marginal and internal gaps in endocrowns made from three different CAD/CAM materials.	30 human molars were divided into 3 groups (*n* = 10): LDGC, resin-modified ceramic, and RNC, and assessed for their accuracy of marginal and internal adaptation of the endocrowns with CBCT.	All tested materials exhibited clinically acceptable marginal gaps (<160 μm). The internal gaps were not clinically acceptable for all materials except for RNC.

Abbreviations: FRC; fiber-reinforced composite, PICN; polymer-infiltrated ceramic network, LDGC; lithium disilicate glass-ceramic, LDS; lithium disilicate, ZLS; zirconia-reinforced lithium silicate glass-ceramic, RNC; resin nano-ceramic, ETT; endodontically treated teeth, CAD/CAM; computer-aided design and computer-aided manufacturing.

A common area of interest among the included studies was the mechanical behavior of endocrowns—particularly fracture resistance—which was commonly evaluated under simulated masticatory conditions. Fifteen articles specifically compared endocrowns with traditional post-and-core crowns [6,8,10,13,19,23,24,27,28,30,31,32,35,38,41], while six articles compared them to other types of restorations, highlighting differences in performance outcomes [1,4,14,19,20,42]. In ten studies, the influence of restorative material on functional durability was investigated by comparing materials such as lithium disilicate, zirconia-reinforced lithium silicate, and resin-based ceramics [2,11,16,20,29,33,34,40,43,44]. Additionally, ten studies examined the decisive role of preparation design on the final performance of endocrowns, evaluating the impact of pulp chamber depth and ferrule presence on the integrity of the final restoration [7,12,15,16,18,36,37,39,43,45]. Marginal adaptation, described as a critical aspect in preventing microleakage and enhancing long-term durability, was appraised in five articles [12,15,16,37,39].

Results from the included studies and systematic reviews are presented in this scoping review. Some primary studies appear in multiple reviews; therefore, reported values may reflect overlapping data. No new statistical pooling was performed, and reported meta-analytic results are presented with their original heterogeneity measures (e.g., I^2^) where available on the corresponding studies [6,9,23].

## 4. Discussion

### 4.1. Key Findings of This Scoping Review

#### 4.1.1. Clinical Indications: When and Why to Choose Endocrowns

Based on the research results, endocrowns are primarily indicated for restoring posterior ETT with substantial structural loss, particularly when post-and-core methods are not feasible [13,30,42]. Therefore, endocrowns are suitable for molars with short, obliterated, calcified, or divergent roots, where conventional posts may pose clinical challenges [2,9,10,12,13,15,18,30,42]. Additionally, they are recommended for restoring teeth where an adequate ferrule effect cannot be achieved and where interocclusal space is insufficient [2,9,10,12,13,18,30,42,46]. Traditional crown preparation may require the removal of 67.5–75.6% of the tooth tissue. In contrast, the decay-oriented design of endocrowns facilitates the preservation of tooth viability [16,39] and there is no need for post-preparation into the root canals, which has been associated with an increased chance of vertical fracture [9]. The micro- and macro-mechanical retention of endocrowns relies on the adhesive cementation and stability derived from an adequate pulp chamber for bonding [8,16]. El Ghoul et al.’s findings correlate with other studies, which indicate that endocrowns provide superior fracture resistance compared to post-and-core crowns due to their single-piece design, which minimizes internal stresses and enhances strength through improved occlusal thickness, while preserving peripheral enamel for better adhesion and load distribution [41].

- Thus, endocrowns are best suited for molars with extensive coronal damage, limitations in root anatomy, or reduced interocclusal space, especially when preserving tooth structure is a clinical priority, as conventional post and core crowns may fail to provide adequate material strength, thickness, and structural support, making endocrowns a more reliable treatment modality.

#### 4.1.2. The Impact of Cuspal Reduction and Intracoronal Extension on Retention and Fracture Resistance

To ensure the longevity of endocrown restorations, efforts have been made over the years to develop proper preparation guidelines and design protocols through numerous clinical and laboratory studies. It is essential to emphasize the potential for preparation and design modifications based on individualized clinical conditions [14]. Most recommendations focus on parameters such as cuspal reduction and occlusal thickness. While no standard line on these parameters has been established, based on the majority of eligible studies, the cuspal reduction ranges between 2 and 3 mm [6,15,16], with Dartora et al. suggesting a slightly broader range of 1.5 to 3 mm [42]. Otto et al. demonstrated that greater occlusal thickness in endocrowns is related to increased fracture resistance, emphasizing the mechanical advantage of thicker restorations [14,35]. While increased occlusal thickness generally enhances fracture resistance, Taha et al. and Mostafavi et al. agreed that aggressive preparations should be avoided, as these could compromise tooth structure without providing desirable clinical benefits [16,47]. Notwithstanding, it is important to consider that other factors may influence cuspal reduction, including tooth type, interocclusal space, cavity depth, remaining axial wall thickness, and the restorative material used [16,20].

- Therefore, an occlusal thickness of approximately 3 mm is generally recommended to optimize fracture resistance but should be carefully modified based on anatomical and material-related factors to prevent excessive tooth reduction.

In general, the occlusal thickness of endocrown restorations typically ranges between 3 mm and 7 mm [20,23,47], including the cuspal reduction and the intracoronal extension [5]. Adequate pulp chamber depth is essential for macro- and micro-mechanical retention [9,10,11,22,27,36]. It is crucial for the preparation not to extend the pulpal floor through the radicular orifices, as a more complex preparation can result in insufficient marginal and internal adaptation, ultimately compromising the long-term performance of the final restoration [5,16,24,27]. Although there is no consensus on the exact chamber depth, Zhang et al. observed that while increasing chamber depth from 1 mm to 3 mm led to higher stress on the restoration, the stress on tooth tissue remained relatively stable, except for a noticeable increase at the root furcation with 3 mm depths, particularly under horizontal loading. It was interesting to note that the 2 mm depth exhibited the best stress distribution, which was the least concentration on the restoration and on the surrounding tooth tissues [48]. This is in agreement with Hayes et al., who found that deep extensions greater than 2 mm were factors that increased the probability for catastrophic failures under oblique forces [49]. Dartora et al., however, demonstrated that the mechanical resistance of the restoration depends on the extent of the pulp chamber extension, which contributes to improved stress distribution of masticatory forces and increased resistance [10,22,36]. They also emphasized that when the pulp chamber depth is only 1 mm, the restoration is prone to rotational motion [10,16,36]. Thomas et al. agree with the fact that very shallow chambers (<2 mm) may lead to debonding, which could lead to reducing the success of the endodontic therapy due to coronal microleakage [9]. Veselinova et al. strengthened this conservative approach by using a 2 mm depth for their investigation and stating that there was no significant difference between 4 mm and 2 mm depths, but deeper extensions tended to fail more seriously [20]. Alqarni et al. found that an intracoronal depth of 2 mm provided greater fracture resistance. However, a 4 mm depth resulted in more catastrophic, non-repairable fractures, while 0 mm showed failure patterns similar to those of untreated teeth [50].

- Overall, these findings suggest that while both underextension and overextension carry risks, a 2 mm pulp chamber depth may provide the best balance between retention, stress distribution, and clinical safety.

The retention of the restoration depends on the surface available for adhesion; thus, smaller pulp chambers lead to higher failure rates and debonding [7,11,13,20,21,23,27]. When endocrowns have a disadvantageous height-to-width ratio, greater leverage forces are generated, and a higher risk of restoration displacement due to adhesive rupture may occur [10,13,23,27]. In the molar region, the orientation and concentration of axial forces play a critical role in the clinical performance of endocrowns [6,23]. Another important factor is the relationship between bone height and the pulp chamber floor, which significantly influences the mechanical behavior of the restoration. Ribeiro et al. [18] demonstrated that when the pulp chamber floor is positioned above the crestal bone level, the risk of mechanical failure increases due to unfavorable stress distribution.

- Clinically, achieving an adequate pulp chamber depth and maintaining a favorable height-to-width ratio are essential to reduce debonding and stress concentrations—particularly in molars and in cases with limited bone support.

#### 4.1.3. Pulp Chamber Cavity Preparation: Balancing Retention, Resistance, and Bond Strength

In addition to considerations related to the pulp chamber cavity, meticulous internal preparation is essential to ensure accurate placement and optimal adaptation of the final restoration. [10,16]. A flat surface floor with an axial wall divergence of approximately 6 degrees, without undercuts but with rounded angles, increases precision in internal fit [5,16,42]. To further optimize clinical outcomes, the immediate dentin sealing (IDS) technique using flowable composite resins can be employed to cover irregularities in the pulp chamber walls [5,10]. This approach not only eliminates retentive zones that hinder the adjustment of the restoration but also improves adhesion to dentin, reduces microleakage, and strengthens the bond in ETT, where dentin adhesion is weaker than enamel [3,5,10,11,30]. Additionally, the cementation protocol plays a critical role in the performance of endocrowns, as optimal adhesion between the restoration and tooth structure directly influences stress distribution and fracture resistance [23]. Inadequate cementation may compromise micromechanical retention, increasing the risk of debonding [5,7]. Resin cements are the material of choice due to their excellent bonding capabilities, color adaptability, adequate mechanical properties, and resistance to dissolution [5]. However, polymerization shrinkage poses drawbacks, including an increased risk of microleakage, resulting in a higher susceptibility to adhesive failure, especially when marginal gaps or insufficient retentive height occur. These conditions aggravate stress accumulation at the tooth-cement interface [7,37].

- Thus, optimal internal preparation, use of immediate dentin sealing, and careful resin cementation are essential to prevent microleakage, improve retention, and minimize stress at the tooth-restoration interface in endocrown restorations.

#### 4.1.4. The Effect of Finish Line Design on Flexural Strength, Stress Distribution, and Internal Adaptation

Another major factor influencing the clinical outcome of endocrown restorations is the finish line design [7,51]. The circumferential 90-degree butt-joint margin, typically 1–2 mm in width, remains the most commonly used configuration. However, several studies have shown a growing preference for shoulder margins and the incorporation of a ferrule effect [5,6,15,16,24,39].

The shoulder margin has been associated with improved flexural strength and stress distribution compared to the butt-joint margin [16,47,51,52]. Incorporating a ferrule—a short axial wall in the cervical area—acts as a bracing mechanism, enhancing both structural integrity and the surface area available for adhesion [1,13,16,23,27,29]. Einhorn et al. [15] reported that a 1 mm ferrule significantly reduced the incidence of irreparable fractures compared to a 2 mm ferrule or no ferrule. Similarly, Taha et al. [47] recommended the preconditioning of a short axial wall with a shoulder finish line to improve mechanical strength. In situations where a ferrule is absent, Mostafavi et al. and Govare et al. [16,27] suggested adding a beveled margin to enhance adhesive potential. Zeng et al. [39] evaluated stress distribution among butt-joint, 90° shoulder, and 135° shoulder designs. While the general stress patterns were similar, the 135-degree shoulder demonstrated lower stress concentration and improved biomechanical viability.

Cervical margin placement should also be carefully considered. Supragingival positioning is preferable [6,10,15,24,27,42], particularly for preserving enamel near the cementoenamel junction, which is essential for optimal bond strength [16,23,27,39]. In such scenarios, the butt-joint margin may be advantageous as a less invasive technique—preserving tooth structure, offering resistance to compressive forces, and reducing marginal leakage [16,39,47]. Taha et al. [47] demonstrated that CAD/CAM polymer-infiltrated ceramic endocrowns—whether with a butt-joint or shoulder margin—can resist forces exceeding typical axial masticatory loads in the molar region, which generally range between 600–900 N [20], with some studies reporting up to 850 N [47]. Interestingly, Einhorn et al. [15] noted that the simpler preparation associated with butt-joint designs may lead to better internal adaptation compared to configurations incorporating a ferrule.

- To sum up, while the butt-joint margin remains a minimally invasive and effective option, shoulder finish lines with short ferrules may enhance fracture resistance and stress distribution. Cervical margin placement should be supragingival whenever possible to preserve enamel and maximize bond strength. Individual anatomical characteristics, enamel availability, and material choice should all be taken into consideration when choosing a margin in clinical practice.

#### 4.1.5. Choosing the Right Material: Mechanical and Esthetic Considerations in Endocrown Performance

The advancement of biomaterials has introduced new indirect restorative materials with an elastic modulus like that of dentin, while simultaneously providing superior fracture resistance. It is well known that the elastic modulus of enamel and dentin is 84.1 GPa and 18.6 GPa, respectively [37]. As the restoration’s physicomechanical characteristics get closer to those of the dentin, the risk of catastrophic fractures decreases [27]. The monoblock design of endocrowns, which minimizes material interfaces, improves stress distribution compared to conventional post-and-core restorations that combine materials with different elastic moduli [9,23]. Several studies have compared lithium disilicate-based ceramics (LD/LDS/LDSB) or lithium disilicate glass ceramics (LDGC) with different types of zirconia (monolithic zirconia, zirconia-reinforced lithium silicate/ZLS), indirect resin-based materials (RB), or resin nanoceramics (RNC) in terms of restoration’s adaptation, bond capacity, fracture resistance, optical and biomechanical properties, biocompatibility, and wear (Table 3).

Lithium disilicate-based ceramics have been considered as the material of choice for endocrown restoration due to their superior adhesive properties and resistance to displacement [2,13,41]. Strong bonding is achieved through micromechanical “interlocking” between the etchable ceramic material and the resin cement, enhancing the stability of the restoration [13,27,36,41]. Additionally, its high aesthetic quality and superior fracture resistance make it a promising choice for dental applications [27]. However, there are several limitations associated with LDS. The high elastic modulus of lithium disilicate ceramics, approximately 90 GPa, often increases the risk of tooth fractures, which could result in irreparable fractures under excessive stress [19]. Furthermore, the material’s stiffness may induce wear on opposing natural teeth, while its brittle nature often results in restoration failure due to porcelain fracture [2,11].

Monolithic zirconia has a modulus of elasticity above 200 GPa, which is significantly higher than that of dentin and thus is prone to catastrophic failures [43]. When Veselinova et al. examined monolithic zirconia and CAD/CAM lithium disilicate endocrowns, they found that the latter exhibited higher fracture strength and less catastrophic failures compared to the former, which mainly fractured beneath the cementoenamel junction [20,53]. When Alwadai et al. examined the lithium disilicate glass ceramics versus zirconia-reinforced lithium disilicates for optimal marginal adaptation, they found that both were within the range of values deemed clinically acceptable [12]. An in vitro study by Kumar et al. showed that endocrowns made from monolithic zirconia exhibited a larger internal gap compared to those made from lithium disilicate (LDS) [54]. In contrast, Falahchai et al. found that zirconia endocrowns exhibited superior marginal and internal fit compared to LDS and ZLS, with the most significant gaps consistently observed in the pulpal area and the smallest at the margins [55].

Zirconia-reinforced lithium silicate (ZLS) ceramics combine zirconia’s mechanical strength and the aesthetic properties of lithium disilicates, offering an improved alternative for endocrown restorations [44]. Jalalian et al., in their in vitro study, demonstrated that ZLS has better margin adaptation and higher fracture resistance than LDS, but they also showed that ZLS has increased amounts of irreparable fractures [43]. On the contrary, El Ghoul et al.’s study demonstrated that LDS exhibits the highest fracture resistance under lateral loading compared to RNC and ZLS, related to its superior adhesive properties and crystalline structure [41]. In adhesive interfaces, lateral forces are more hazardous than axial ones, because stresses are concentrated in the cervical region rather than distributed along the long axis, increasing the risk of irreparable fractures [20,41]. However, the high elastic modulus of lithium disilicate (LDS, 95 GPa) exerts greater stress on weaker surfaces, increasing the risk of irreparable fractures, whereas more flexible materials like resin nano-ceramics (RNC, 20 GPa) and zirconia-reinforced lithium silicate (ZLS, 70 GPa) distribute stress more homogeneously [41]. Manziuc et al. reported that zirconia-reinforced lithium silicate (ZLS) exhibited superior mechanical properties compared to feldspathic ceramics, lithium disilicate ceramics, and resin nano-ceramics, but demonstrates inferior esthetics due to the presence of tetragonal zirconia particles [44].

Resin-based materials, including resin nanoceramics (RNC), polymer-infiltrated ceramic networks (PICN), and millable composite resins, are emerging as reliable alternatives for endocrown restorations [2,19,38]. PICN consists of a 25% by volume polymer network and a 75% by volume ceramic network that combines the long-term aesthetic stability of ceramics with the preferred elastic modulus of resin composites [2,56]. RNC are composed of a resin-based matrix with dispersed fillers (nanoceramic particles), with the exact composition varying according to the specific product [2]. The materials exhibit high fracture resistance and an elastic modulus similar to that of dentin (18.6 GPa), thereby preventing catastrophic fractures. They tend to deform more before failing by changing the stress distribution and the contact with the surface of the restorative assembly [2,27,40,42]. Dartora et al. showed that PICN offers a comparable fatigue failure load to LD restorations over the same number of loading cycles [42]. Beji Vijayakumar et al. concluded that RNCs have increased resilience to fractures compared to ZLS, although ZLS outperforms PICN in fracture resistance [2]. Keskin et al. reported survival rates of 82.7% for RNC and 86.8% for ZLS over a 3-year evaluation period, with no statistically significant difference between the two materials [33]. Resin-based materials experience fewer catastrophic failures, especially under axial loading, since they have the ability to absorb and distribute stresses homogeneously. Furthermore, these materials can be easily repaired intraorally [2]. Although their lower modulus of elasticity may reduce stress within the dentin, it can increase stress at the adhesive interface, thereby raising the risk of debonding [2,19,27,42]. The success of restorations depends on both internal and marginal fit, which is influenced by the material used. Taha et al. found that while LDGC, RNC, and resin-modified ceramics showed acceptable margin gaps, only RNC achieved a clinically acceptable fit [45].

**Figure 3:**
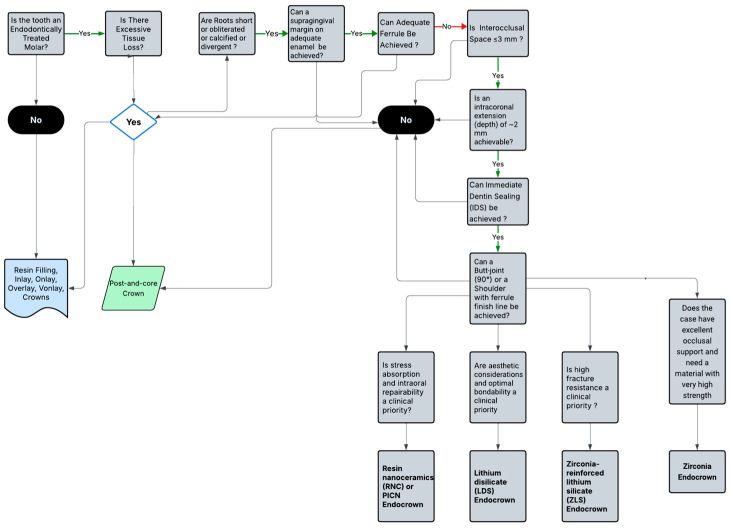
Clinical decision tree for endocrown restoration selection.

To support clinical decision-making in the restoration of endodontically treated posterior teeth, a decision tree was developed based on the findings of this scoping review (Figure 3). This visual guide summarizes key criteria for selecting endocrown restorations, including remaining tooth structure, pulp chamber depth, presence of ferrule, available interocclusal space, material choice (LDS, ZLS, RNC), and adhesive technique. The aim is to assist clinicians in making evidence-based, case-specific restorative decisions that enhance the predictability and long-term success of treatment outcomes.

#### 4.1.6. CAD/CAM-Fabricated Endocrowns: Precision, Efficiency, and Material Compatibility

The continuous use of CAD/CAM technology in restorative dentistry for milling endocrown restorations enables chairside treatment in a single appointment [37,57]. One of the main advantages demonstrated is the superior fracture resistance of CAD/CAM all-ceramic endocrowns, which surpasses that of non-CAD/CAM all-ceramic restorations [4,11,37]. According to Kuang et al. CAD/CAM all-ceramic endocrowns had a 5-year survival rate of 93.0% [34]. Fages et al. in their seven-year clinical trial observed a 98.66% survival rate of chairside CAD/CAM feldspathic ceramic endocrowns [31]. Similarly, Otto and Morman’s 12-year clinical study on chairside CAD/CAM feldspathic ceramic endocrowns revealed high survival rates, with 90.5% for molars and 75% for premolars [35].

- Even though CAD/CAM endocrowns have extremely high survival rates and seem a successful long-term alternative, careful case selection and proper bonding techniques are essential to minimize risks and ensure successful outcomes.

### 4.2. Survival and Success Rates: Long-Term Outcomes and Clinical Predictability of Endocrowns

In terms of survival and success rates, endocrowns and post-and-core crowns appear to offer comparable outcomes for restoring endodontically treated molars [1,3,6,12,16,27,32]. Studies have shown that endocrowns demonstrate similar or even greater survival rates under repeated and static loads, generally due to their distinctive design and adhesive bonding mechanism, which promote better stress distribution and load transmission [4,23,28]. For instance, Fathi et al. reported a 5-year survival rate of 91.4% for endocrowns and 98.3% for post-core crowns, with no significant clinical differences [32], while Al-Dabbagh et al. demonstrated a 5-year survival rate of 89.1% for endocrowns and 98.2% for conventional crowns in molars [6]. In a 4-year retrospective study by Ayata et al., endocrowns demonstrated complete survival during the follow-up period [58]. Belleflamme et al. highlighted impressive survival rates of up to 99% for endocrowns in posterior teeth, with Qamar emphasizing that this is the only long-term study providing a 10-year survival rate over such an extended period [24,30]. Govare et al. mentioned that endocrowns had only 6% of root fractures, and 71% of failures were due to loss of retention, whereas the root fracture rate for post or no post crowns was at 29% [27].

- The success rate of endocrowns varies across different studies but consistently remains high, making them an excellent conservative option for restoring endodontically treated teeth.

### 4.3. Limitations of This Scoping Review

While this scoping review provides a broad overview of the available literature on endocrown restorations in endodontically treated molars, it is important to acknowledge several limitations associated with its methodological framework. Firstly, unlike systematic reviews, scoping reviews typically do not include an assessment of the quality of the eligible studies or an evaluation of the risk of bias using critical appraisal tools. As a result, the reliability and internal validity of the included sources were not critically appraised, which limits the ability to draw definitive conclusions or make evidence-based clinical recommendations. Some studies included in multiple systematic reviews may contribute overlapping data. As a scoping review, this descriptive synthesis does not re-pool data; therefore, readers should interpret the reported quantitative outcomes with caution. Additionally, although comprehensive searches were conducted in two major databases (PubMed and Scopus) and reference lists were thoroughly screened, other electronic databases such as Embase, Web of Science, and Cochrane CENTRAL were not searched due to resource limitations.

Despite screening reference lists to minimize omissions, relevant grey literature and unpublished data may have been missed. The variability in study designs, outcome measures, and reporting standards posed a significant challenge for data synthesis and meaningful comparison. Excluding case reports, conference abstracts, and non-English publications may have omitted early, preliminary, or non-English evidence, which could limit the overall comprehensiveness of the review and introduce potential language bias. These limitations highlight the need for future systematic reviews and meta-analyses to provide more definitive guidance based on rigorous appraisal and quantitative synthesis.

#### Methodological Limitations of the Included Studies

The included studies present several methodological limitations. Most investigations were in vitro, which may not precisely replicate clinical conditions. Sample sizes were often small, constraining the statistical power of the results. Direct comparisons were also challenging due to the significant heterogeneity across studies in terms of restorative materials, loading protocols, preparation designs, and evaluation techniques. These limitations should be taken into consideration when evaluating the findings and applying them in clinical practice.

### 4.4. Clinical Implications and Recommendations

- Indications: Endocrowns are best suited for posterior ETT, particularly molars with short, calcified, or divergent roots where post-and-core restorations are not feasible.
- Case selection: Avoid endocrowns in teeth with insufficient pulp chamber depth (<2 mm), limited enamel margins, or unfavorable height-to-width ratios.
- Pulp chamber depth: A 2 mm extension into the pulp chamber is generally recommended, as it balances retention and stress distribution while minimizing catastrophic failures.
- Occlusal thickness: Maintain around 3 mm occlusal reduction for optimal fracture resistance, but avoid unnecessary removal of tooth structure.
- Ferrule and finish line: A 1 mm ferrule with shoulder finish line improves stress distribution and reduces irreparable fractures; if not possible, a butt-joint margin is a conservative alternative.
- Preparation design: Ensure a flat pulpal floor, 6° divergence, and rounded internal angles, and consider immediate dentin sealing (IDS) to enhance adhesion.
- Material selection:
  1. LDS: Strong adhesive bond and esthetics, first choice when high strength and reliability are needed.
  2. ZLS: Combines good strength and moderate esthetics.
  3. RNC/PICN: More elastic and repairable, reducing catastrophic fractures, but higher risk of debonding.
- Cementation: Resin cements are preferred, ensuring adequate adhesion to minimize debonding and stress accumulation.

## 5. Conclusions

This scoping review provides a comprehensive synthesis of the current literature on the use of endocrowns for restoring extensively damaged posterior endodontically treated teeth. Based on the available evidence, endocrowns are particularly indicated in cases of significant coronal tooth structure loss, limited interocclusal space, and root canals with complex or atypical morphology. Notably, the presence of enamel along the majority of the restoration margins is a critical factor for ensuring long-term clinical success. Additionally, endocrown restorations are time-efficient, involving fewer procedural steps, reduced chairside time, and overall lower treatment costs compared to conventional post-and-core restorations. Furthermore, the use of resin-based materials and lithium disilicate ceramics has emerged as the most preferable choice, owing to their advancements in fracture resistance and aesthetic properties.

Multiple parameters influence the clinical and laboratory performance of endocrown restorations in endodontically treated teeth (ETT). Among others, the amount of the remaining tooth structure, the presence or absence of a ferrule, and the tooth’s location within the dental arch are the most critical factors for choosing a specific type of restoration. Despite growing clinical acceptance, there is a continued need for standardization in both preparation protocols and material classification. The current literature reveals significant heterogeneity, primarily due to inconsistent descriptions of tooth preparation techniques for endocrowns. While short-term findings are encouraging and support endocrowns as a viable alternative to conventional post-and-core restorations, the lack of robust long-term evidence limits definitive conclusions regarding their superiority in molars.

Clinical Recommendations:
  - Endocrowns are best suited for molars with extensive coronal loss, short roots, or limited interocclusal space.
  - Preservation of enamel at the restoration margins is essential for optimal adhesion and long-term success.
  - Lithium disilicate or resin-based materials should be preferred due to their superior fracture resistance and esthetics.

Future Research:

Further well-designed randomized clinical trials with long-term follow-up are necessary to resolve the discrepancies and establish standardized preparation protocols, clarify the role of pulp chamber depth and ferrule design, and compare the clinical outcomes of different restorative materials.

## Figures and Tables

**Table 1:** Systematic reviews and meta-analyses on endocrown restorations.

Author(s)/Publication Year	Aim	Materials and Methods	Conclusion
**Comparison of Endocrown Restorations to Conventional Crowns**			
[23] Sendrez-Porte et al., 2016	Systematic review of clinical and in vitro studies comparing endocrown restorations with conven [tional treatments (posts-and-core crowns, composite resin, inlays/onlays), testing the hypothesis that endocrowns perform similarly.	Pubmed (MedLine), Lilacs, Ibecs, Web of Science, BBO, Scielo, and Scopus Identified records: 103 Included records: 8 published between 1999 and 2014	Endocrowns offer comparable or superior performance to traditional methods, such as posts-and-core crowns, with demonstrated high success rates (94–100%), greater fracture strength, and favorable biomechanical performance, particularly in terms of failure patterns. Further studies are needed to validate long-term outcomes.
[27] Govare et al., 2020	Evaluation of endocrowns as a restorative option for extensively damaged teeth, assessing their predictability, success, survival rates, and fracture strength compared to post-retained restorations.	PubMed, Scopus, Embase, and the Cochrane Library Identified records: 110 Included records: 41 published between 1999 and 2018.	Endocrowns offer a reliable alternative to post-retained restorations, especially for molars, with comparable or superior fracture strength.
[6] Al-Dabbagh et al., 2021.	A review and analysis of the survival and success rates of endocrowns vs. conventional crowns, offering evidence—based guidance for restoring extensively damaged teeth.	PubMed, Scopus, EMBASE, Cochrane, and Google Scholar (up to June 2019) Identified records: 2584 Included records: 10 articles for systematic review and 3 clinical studies for meta-analysis.	Endocrowns showed a 5-year survival rate of 91.4% and a success rate of 77.7%, compared to 98.3% and 94% for conventional crowns, with no significant differences (*p* > 0.05).
[24] Qamar et al., 2023	Comparison of the physical and mechanical properties of LDS endocrowns on posterior teeth and post-and-core restorations.	PubMed, Embase, Scopus, ISI Web of Knowledge (WoS), Google Scholar, unpublished studies, conference proceedings, and cross-references up to 31 January 2023 Identified records: 291 Included records: 10	There is no significant difference between the fracture strength and failure rates of LDS endocrowns and conventional post-and-core crowns.
[28] Lenz et al., 2024	Comparison of the biomechanical performance of endocrowns and traditional post-and-core crown restorations (with and without intracanal posts) for rehabilitating ETT with severe coronal structure damage.	MEDLINE/PubMed, Scopus, and Web of Science, based on in vitro studies Identified records: 291 Included records: 31 published between 2015 and 2023.	Endocrowns exhibited similar or greater biomechanical performance than post-and-core crown restorations across most evaluated studies. Demonstrated favorable survival rates under fatigue and monotonic loads, lower stress levels in restorative materials, and better failure patterns compared to post-and core-crowns.
[29] Matos et al., 2024	Assessment of clinical performance (survival rate, failure risk, fracture incidence) and laboratory outcomes (fracture mode, failure analysis) of rehabilitated ETT.	Pubmed, Scopus, Web of Science, Embase, Cochrane, Open Grey, and manually Identified records: 89 articles Included records: 38 (31 in vitro and 7 RCTs)	No significant difference in fracture resistance or failure modes between ETT with or without posts. Survival rates were similar, though failure risk was lower with posts. The need to consider tooth characteristics and remaining structure for each case.
**Material-Based Comparison**			
[2] Beji Vijayakumar et al., 2021	Evaluation of whether RB endocrowns exhibit better fracture resistance and fewer catastrophic failures compared to LDS endocrowns in vitro studies.	PubMed, EBSCOhost, Cochrane Central Register of Clinical Trials, Google Scholar, and manually. Identified records: 229 Included records: 5 published between 2015 and 2020.	RB endocrowns demonstrated similar or higher fracture resistance under axial forces and fewer catastrophic failures compared to LDS endocrowns.
[12] Alwadai et al., 2023	Analysis of in vitro studies on marginal adaptation of CAD/CAM and heat-pressed LDS and ZLS endocrowns.	Web of Science, PubMed, EMBASE, Scopus, Cochrane, Google Scholar, and ProQuest Identified records: 428 Included records: 17 published between 2016 and 2023.	All-ceramic LDGC and zirconia endocrowns for posterior teeth, fabricated via CAD/CAM or heat-press, showed acceptable marginal adaptation.
[11] AlHelal et al., 2024	Assessment of fracture resistance of CAD/CAM vs. non-CAD/CAM endocrowns.	Embase, Web of Science, and Scopus. Identified records: 1591 Included records: 17	CAD/CAM endocrowns show superior fracture resistance compared to non-CAD/CAM options.
**Survival and Success Rates**			
[7] Papia et al., 2020	A review of the literature on endocrowns, focusing on success, survival rates, and how designs, materials, and cements influence outcomes, provides guidance for restoring extensively damaged teeth.	Searches in PubMed, Cochrane, and Scopus Identified records: 3472 Included records: 6 Published between 1999 and 2017.	Feldspathic endocrowns with a 1–4 mm pulp cavity, 1–2 mm shoulder preparation, and adhesive resin cement show promise for molars.
[16] Mostafavi et al., 2022	Evaluation of how preparation designs affect marginal integrity and fracture resistance of endocrowns, aiming to identify optimal designs for restoring severely damaged teeth.	Searches in PubMed, Embase, Scopus, and the Cochrane Library Identified records: 200 included records: 16 published up to February 2021.	Endocrown preparation design impacts marginal adaptation and fracture resistance, with excessive preparation reducing performance and increasing non-repairable fractures. Simpler cavity configurations are recommended.

Abbreviations: LDGC; lithium disilicate glass-ceramic, LDS; lithium disilicate, ETT; endodontically treated teeth, CAD/CAM; computer-aided design and computer-aided manufacturing, RCT; randomized clinical trials.

**Table 3:** Comparison of endocrown materials.

Material	Elastic Modulus	Fracture Resistance	Esthetics	Bonding	Failure Type	Recommended Application
LDS	~90–95 GPa	Excellent	Excellent	Strong micromechanical bond	Often catastrophic	High-esthetic zones, molars with deep chambers
ZLS	~70 GPa	Very good	Moderate–High	Good	Mixed failures (some catastrophic)	Balanced cases with moderate esthetic needs
RNC	~20 GPa	Moderate–High	Moderate	Weaker than LDS	Mostly restorable, flexible, debonding at adhesive interface	Bruxism, minimal preparations, repairable restorations intraorally
PICN	~30 GPa	Moderate	Moderate	Moderate	Deformable, restorable, debonding at adhesive interface	Patients with parafunctional activity or low occlusal clearance
Monolithic Zirconia	>200 GPa	High but brittle	Low–Moderate	Weak	Catastrophic, root fracture	Not commonly recommended, low-esthetic/high-load areas only, good marginal fit

**Figure A1:**
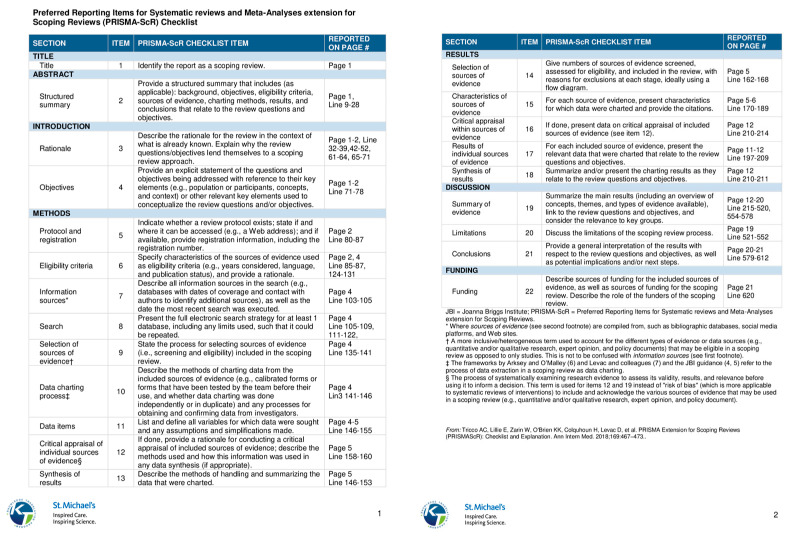
The Prisma Check list.

**Figure A2:**
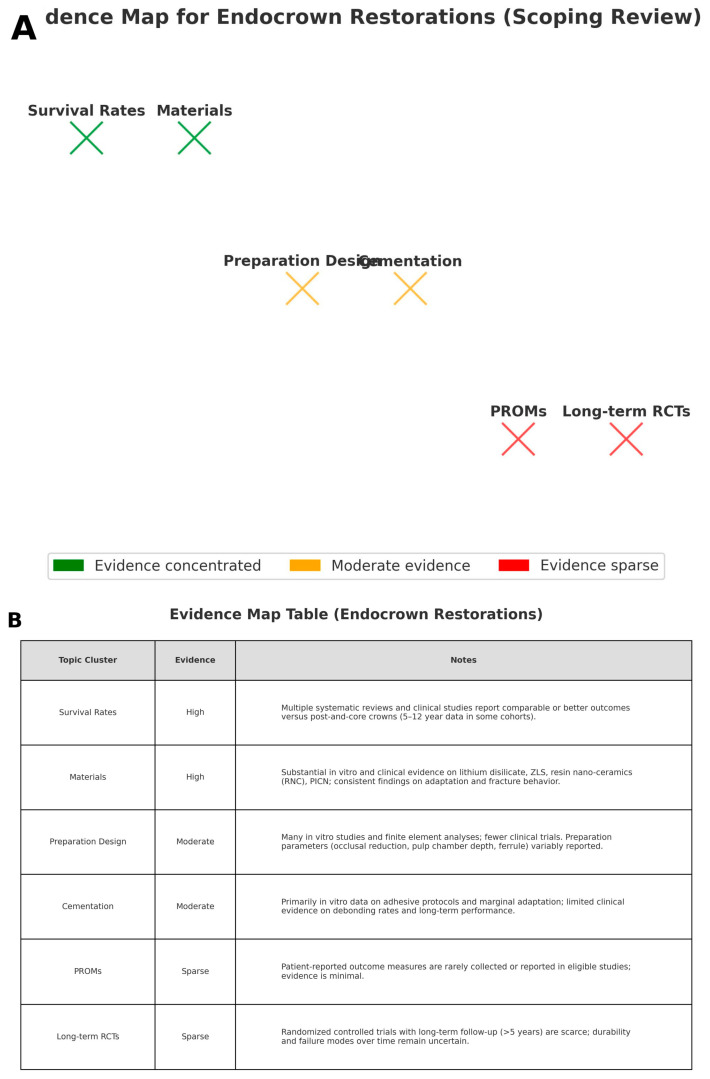
(**A**) Visual evidence map showing areas where research is concentrated (green = abundant evidence; orange = moderate evidence; red = sparse evidence). Evidence is strongest for survival rates and restorative materials, moderate for preparation design and cementation, and sparse for patient-reported outcome measures (PROMs) and long-term randomized controlled trials (RCTs). (**B**) Evidence summary table providing descriptive notes for each topic cluster, detailing the nature and scope of the available studies.

## Data Availability

Data sharing is not applicable to this article as no datasets were generated or analyzed.

## Supplementary Materials

The following supporting information can be downloaded at: https://www.prisma-statement.org/scoping (accessed on 10 July 2025), PRISMA-ScR Checklist.

## Appendix A

The final electronic search was conducted in PubMed (MEDLINE) and Scopus on 6 July 2025 (date stamp). The search was restricted to publications up to March 2025, applied in two separate ranges: up to 2024 and January to March 2025. An English language filter was subsequently applied. After screening, a total of 17 articles were excluded.

**Table A1:** Number of Articles Retrieved by Database and Keyword (Publications up to March 2025).

Databases/Keywords	Endocrowns	Endocrowns and Materials	Endocrowns and Ferrule Effect	Endocrowns and Survival Rate	Endocrowns and Post-and-Core Restorations	Total
**PubMed (MEDLINE)**	343	274	38	27	61	743
**Scopus**	315	264	39	32	41	691

## Appendix B

This appendix presents the completed PRISMA-ScR checklist, corresponding to the sections of our scoping review, ensuring compliance with the recommended reporting standards. Each item is matched with the page/line numbers in the manuscript where it is addressed.
